# Polymeric soft materials with molecular recognition: from static binding to dynamic functions in gels, particles, and interfaces

**DOI:** 10.1080/14686996.2026.2664156

**Published:** 2026-05-07

**Authors:** Takashi Miyata

**Affiliations:** a Department of Chemistry and Materials Engineering, Kansai University, Suita, Japan; b Organization for Research and Development of Innovative Science and Technology, Kansai University, Suita, Japan

**Keywords:** Hydrogels: particles: microgels, nanogels, membranes, layers, molecular recognition, molecular complex, stimuli-responsive polymer, molecular imprinting

## Abstract

Molecular recognition governs important chemical events in biology from enzyme catalysis to immune defense and cellular signal transduction, where specificity is expressed not only as affinity but also as regulated, state-dependent function. Translating these principles into synthetic materials and systems has stimulated the development of polymeric soft materials in which binding events are coupled to macroscopic responses. This review focuses on polymeric soft materials such as hydrogels, particles, and interfaces that integrate recognition moieties, including noncovalent and host-guest interactions, biomolecular ligands, and molecularly imprinted cavities, and describes coupling mechanisms that convert binding into macroscopic structural change, transport regulation, and device-readable signals. In macroscopic stimuli-responsive hydrogels, molecular recognition can induce changes in effective crosslink density, hydration, and chain conformation, thereby enabling autonomous gating and representative concepts for glucose-responsive insulin delivery. Miniaturization to particles and micro/nanogels increases accessible surface area and shortens diffusion paths, which often accelerates response kinetics and enhances targeted therapeutics and controlled drug release. At polymer interfaces, especially membranes, thin films, and layers, molecular recognition must be designed together with transport pathways. Antifouling architectures, including zwitterionic polymers, are also important for maintaining selectivity in surface-sensitive measurements such as surface plasmon resonance (SPR). Across these platforms, practical performance is frequently governed not by binding affinity alone but by transport accessibility, layer thickness, and baseline stability against nonspecific adsorption and signal drift. Focusing on the relationships between structures and functions, this review summarizes current progress and outlines design strategies for smart polymer materials that translate molecular binding into functional outputs.

## IMPACT STATEMENT

This review highlights design rules that couple molecular recognition to macroscopic outputs across hydrogels, micro/nanogels, and interfaces, using dynamic crosslinks, miniaturization, and antifouling coatings to achieve high selectivity and responsiveness.

## GRAPHICAL ABSTRACT

**Figure:**
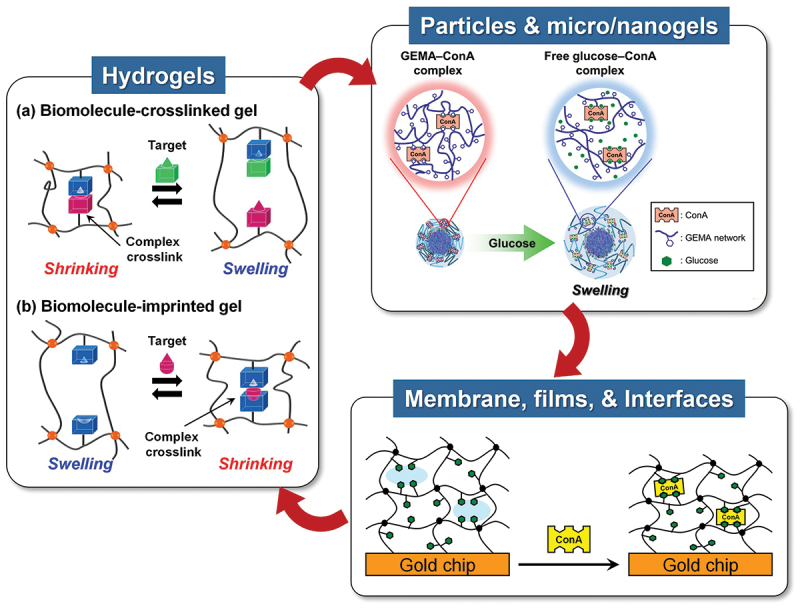

## 1. Introduction

Molecular recognition arises from the cooperative sum of weak interactions, such as electrostatic interactions, hydrogen bonding, hydrophobic interactions, and shape complementarity, providing strong affinity and high selectivity between a host and a guest. In biology, such recognition is central to enzymatic reactions, immunological defense, genetic information transfer, and cellular signal transduction. Classical concepts such as Fischer’s ‘lock-and-key’ model [1] and Pauling’s molecular complementarity theory [2] explain an essential aspect of specificity by emphasizing three-dimensional structural matching between receptors and ligands. However, many protein receptors operate on conformational ensembles rather than fixed structures. Their intrinsic dynamics enable regulatory mechanisms such as allostery, where molecular binding at one site induces conformational changes and modulates activity elsewhere. For instance, the binding of oxygen to hemoglobin, which is a representative allosteric protein, induces a quaternary structural change that increases the affinity of remaining subunits. The allosteric regulation of hemoglobin is a cooperative effect vital for efficient oxygen transport. Mimicking not only the static binding affinity but also such dynamic allosteric function in synthetic materials remains a grand challenge.

Although proteins offer excellent specificity, their widespread use in industrial and clinical settings is often limited by low stability, high production costs, and sensitivity to non-physiological environments [3–7]. These limitations have motivated biomimetic design of synthetic recognition systems, such as crown ethers, cryptands, and related host-guest complexes [8–10], leading to the establishment of supramolecular chemistry. These small-molecule concepts mimicking the ‘lock-and-key’ model were expanded to molecular imprinting, which allows the structure of a target molecule to be memorized within polymeric materials. Molecular imprinting is now widely used as a useful method for the facile and tailored creation of molecular binding sites. The molecular imprinting is classified into two approaches, i.e. covalent and noncovalent approaches. The covalent approach, proposed by Wulff et al. [11], enables the formation of cavities with precise functional group orientation *via* reversible chemical bonds, such as boronate esters, which are cleaved after polymerization. The covalent approach offers high selectivity but requires prior synthesis of template-monomer conjugates. In contrast, the noncovalent approach, proposed by Mosbach et al. [12,13], uses noncovalent interactions between functional monomers and template molecules before polymerization. After the polymerization of the functional monomers around a template molecule, highly specific, complementary binding cavities can be formed within a crosslinked matrix by the template removal. The noncovalent approach becomes the standard for preparing molecularly imprinted polymers (MIPs) due to its operational simplicity and wide applicability to various templates, including drugs and proteins. The molecular imprinting enables synthetic polymers to achieve binding constants (*K*_a_) comparable to those of natural antibodies, offering robust and cost-effective alternatives for affinity separation and sensing. However, while traditional MIPs exhibit static binding for a target molecule, they generally lack the ability to respond to the binding event.

**Figure 1.**
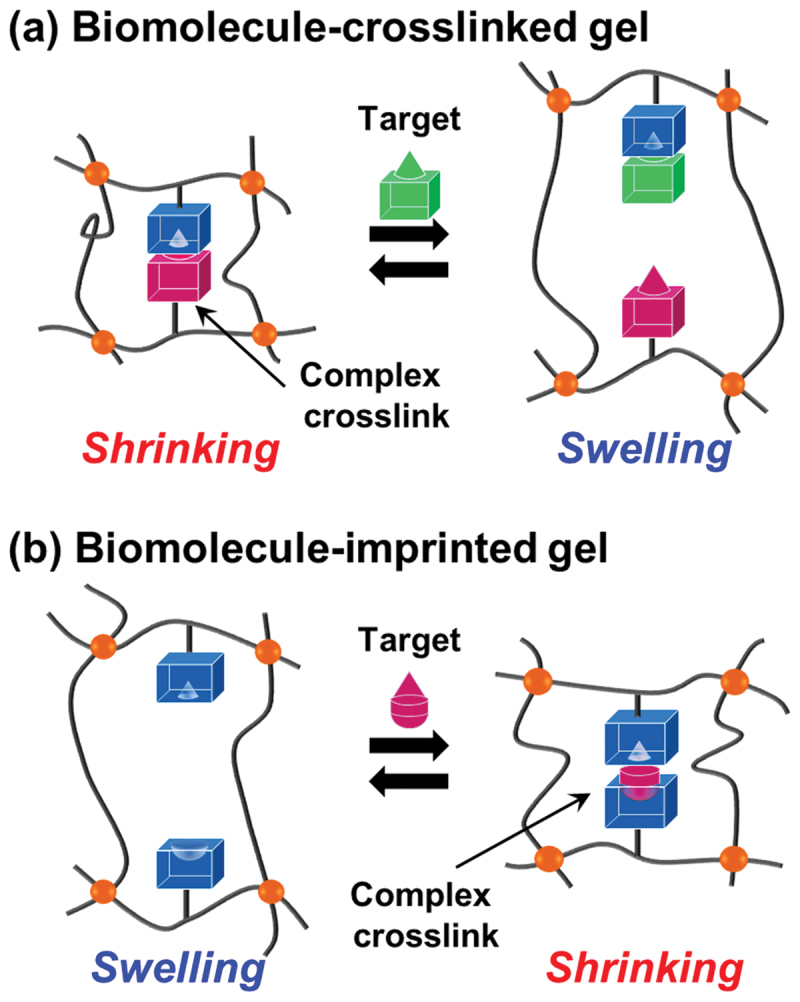
Schematic of swelling/shrinking behavior of biomolecularly stimuli-responsive hydrogels with biomolecular complex crosslinks: (a) biomolecule-crosslinked hydrogel; (b) biomolecule-imprinted hydrogel.

In recent decades, the focus of molecular recognition has shifted from static recognition to dynamic function. For example, the strategic integration of molecular complexes, such as host-guest interactions, antigen-antibody bindings, and DNA duplexes, into stimuli-responsive polymer networks allows for the design of biomimetic polymer materials. The tunable properties of stimuli-responsive polymers are effectively combined with the high selectivity of biological molecules and systems. The regulation of molecular recognition and binding capability can be achieved by introducing ligands and forming molecular binding sites within stimuli-responsive polymer materials. Importantly, the polymer materials with molecular complexes not only bind a target molecule, but also sense it, process the information, and respond *via* macroscopic structural change, mechanical actuation, or signal transduction. For example, bioconjugated hydrogels with biomolecular complexes as dynamic crosslinks can exhibit swelling or shrinking in response to a target biomolecule because of changes in their crosslinking density (Figure 1) [14,15]. In these bioconjugated hydrogels, the molecular recognition event acts as a biochemical trigger that induces localized shifts in cross-linking density, polymer chain conformation, or hydration state. This combination of molecular recognition and responsive behavior translates the free energy of binding into macroscopic functional outputs, such as reversible volume changes or altered optical properties, closely mimicking the allosteric regulation found in native biological systems.

The macroscopic form and dimension of the polymer materials with molecular recognition strongly influence their utility and performance across specific applications. Bulk gel materials, particularly highly hydrated hydrogels, provide a tissue-like three-dimensional microenvironment that facilitates rapid aqueous diffusion while preserving the native conformation of conjugated biomolecules [16–19]. Reducing the material dimensions to the micro- and nanoscale to form polymeric particles dramatically increases the specific surface area, thereby enhancing binding kinetics. In particular, molecularly imprinted polymer (MIP) particles with micro- and nanoscales have been designed as nanomedicine platforms to achieve prolonged circulation, active targeting, controlled drug release, and antitumor efficacy [20]. Furthermore, formulating the polymer materials with molecular recognition as membranes, thin films, or layers allows for seamless integration into solid-state devices and continuous flow systems [21–23]. Such membranes, thin films, and layers provide the essential active interfaces required for intelligent transport gating and localized binding events. In fabricating sensors, these polymeric interfaces with molecular recognition sites are integrated into diagnostic devices, such as surface plasmon resonance (SPR) sensor chips. In responsive polymer films, structural changes induced by molecular binding events are transduced into measurable optical or electrical signals, enabling real-time detection of analytes in complex biofluids [21–23]. Similarly, in targeted drug delivery systems (DDS), molecular recognition is used not only for the specific accumulation of therapeutic carriers at diseased tissue sites but also for triggering the localized release of pharmaceutical drugs in response to precise molecular signals, such as enzymatic activity or inflammatory markers [24–26]. Conversely, the concept of converting specific molecular binding events into macroscopic mechanical responses has even been extended to the self-assembly of macroscopic building blocks driven by host-guest recognition [27].

This review provides structural design and applications of polymer soft materials with molecular recognition, focusing on the progression from static binding to dynamic function. By thoroughly exploring the fundamental principles of biomimetics, molecular imprinting, and biological ligand conjugation across diverse material architectures, including hydrogels, particles, and interfaces, this review highlights the structure-function relationships governing their molecular recognition and responsive behavior. This review also introduces the recent practical applications of these polymer materials with molecular recognition in biosensing, targeted therapeutics, and drug delivery, offering their future perspectives.

## 2. Molecularly stimuli-responsive hydrogels

### 2.1. Glucose-responsive hydrogels

Hydrogels serve as widely used soft material mimics of biological tissues due to their high water content, porous structure, and mechanical properties similar to the extracellular matrix. Some hydrogels, called stimuli-responsive gels, undergo changes in volume in response to environmental changes in pH, temperature, and so on [28–30]. While traditional stimuli-responsive hydrogels exhibit swelling/shrinking depending on non-specific triggers such as temperature or pH, their next generation possesses molecular recognition sites within their polymer networks and responds to target molecules as specific triggers [14,31–35]. Biomolecules such as saccharides and proteins detect defined chemical signals as inputs, ranging from ions and small metabolites (e.g. glucose) to enzymes and nucleic acids, and convert these recognition events into appropriate physiological outputs. Inspired by such signal-responsive biosystems, molecule-triggered polymer materials, i.e. molecularly stimuli-responsive hydrogels that exhibit analyte-induced volume changes, have been prepared as useful tools for designing smart drug delivery systems (DDS) and diagnostic systems. Although most studies have focused on pH- and temperature-responsive polymer materials for a long time, molecularly stimuli-responsive hydrogels, microgels, and nanogels are increasingly emphasized because they can change physicochemical structures and properties, such as hydration, permeability, and network conformation, upon binding or reacting with specific molecules as inputs [15]. As molecularly stimuli-responsive hydrogels can transduce the chemical energy of specific binding directly into macroscopic changes in the structures and properties, they are regarded as attractive candidates for biosensing and site-selective therapy. Thus, molecularly stimuli-responsive hydrogels have many potential applications in designing drug delivery systems, sensors, and cell culture systems.

For diabetes treatments, the development of a self-regulating insulin delivery system, i.e. artificial pancreas, is a critical and long-standing goal. A glucose-responsive drug delivery system as an artificial pancreas must sense blood glucose levels and release insulin precisely when needed, preventing both hyperglycemia and hypoglycemia. Thus, as diabetes treatments with low stress require self-regulating insulin delivery systems, glucose-responsive hydrogels have been investigated as a representative class of biomolecularly stimuli-responsive polymer materials for diabetes treatments for a long time. In diabetes, dysfunction of the pancreatic islets of Langerhans leads to insufficient insulin secretion, necessitating insulin administration in response to the blood glucose level. This clinical requirement has motivated the development of glucose-responsive gel systems that can self-regulate insulin release in accordance with glucose concentration, thereby serving as the basis for therapeutic devices for diabetic patients [34–36]. The earliest studies on glucose-responsive gels used membrane systems that coupled the enzymatic activity of glucose oxidase (GOx) with the pH sensitivity of amino-functional polymer networks composed of *N,N*-diethylaminoethyl methacrylate (DEA) and 2-hydroxypropyl methacrylate (HPMA) [37,38]. Under elevated glucose conditions, GOx entrapped in the DEA-HPMA gel membrane catalyzed the oxidation of glucose to gluconic acid, which decreased the local pH inside the membrane. The resulting protonation of pendant amino groups increased osmotic swelling pressure, inducing membrane swelling and thereby promoting insulin permeation in a glucose-dependent manner.

Early efforts utilized lectin (ConA), which is a tetravalent protein that binds specifically to glucose and mannose, to develop glucose-responsive insulin release systems [39]. This work demonstrated that ConA is a useful tool for designing glucose-responsive systems. This concept was advanced to the use of ConA as a glucose-recognizable moiety. For example, glucose-responsive hydrogels were prepared by immobilizing ConA within networks containing pendant glucose groups, which were formed by polymerization of 2-glucosyloxyethyl methacrylate (GEMA) [15, 40–42]. In the absence of free glucose, as ConA bound to the pendant glucose groups of GEMA, the GEMA-ConA complex acted as a multifunctional crosslinker to keep the hydrogel in a shrunken state. When glucose concentration increased, the free glucose diffused into the gel and competed for the ConA binding sites. Because ConA bound to free glucose by the complex exchange mechanism, the GEMA-ConA crosslinks dissociated, followed by gel swelling. Covalently immobilizing ConA to the network led to reversible changes in the gel volume in response to glucose concentration. Thus, the GEMA-ConA complex is a useful tool as a dynamic and reversible crosslink for designing glucose-responsive hydrogels.

**Figure 2.**
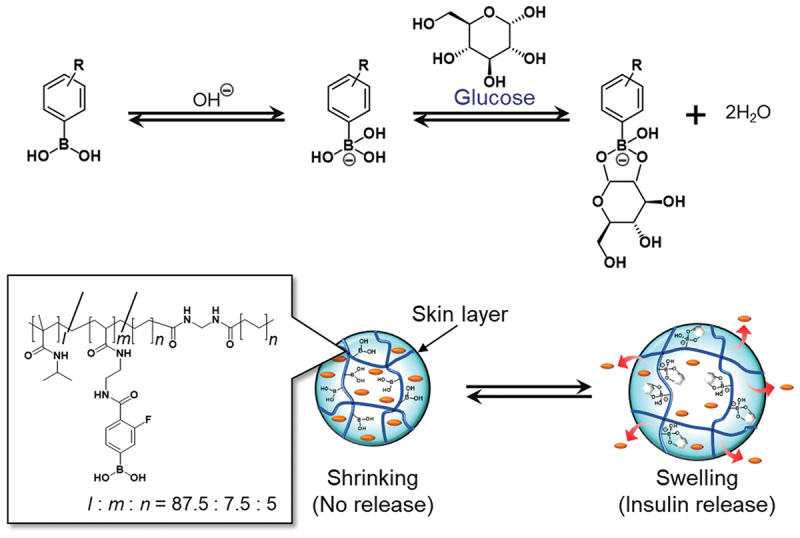
Pancreas-like self-regulated insulin release behavior from a synthetic glucose-responsive gel composed of phenylboronic acid moieties and temperature-responsive polymer chains [48].

To avoid the potential immunogenicity and instability of biological molecules like GOx and lectins, totally synthetic systems have been developed using phenylboronic acid (PBA) as a glucose-binding moiety [43,44]. PBA forms a reversible covalent complex with *cis*-diol compounds like glucose. Kataoka *et al*. developed a glucose-responsive insulin release system by combining the glucose-recognition function of PBA and the temperature-responsive behavior of poly(*N*-isopropylacrylamide) (PIPAAm) and their derivative gels [45–47]. Under normoglycemic conditions, the hydrophobic gel is collapsed, forming a dense skin layer that prevents insulin release. Under hyperglycemic conditions, however, glucose binding to PBA induces the ionization and enhances the hydrophilicity of the polymer chains. As a result, the gel undergoes a phase transition from a shrunken state to a swollen state in response to glucose, disrupting the skin layer and allowing insulin to diffuse out. This reversibly glucose-responsive on-off regulation mimics the pulsatile release of the pancreas. In addition, Matsumoto *et al*. developed a protein-free, PBA-based synthetic glucose-responsive gel that converts interstitial glucose fluctuations into reversible hydration changes, thereby enabling glucose-responsive insulin delivery without electronics in diabetic mice [48]. By integrating this gel into a single catheter-confined device and implanting it subcutaneously, they established a closed-loop concept in which continuous glucose sensing is coupled to gel surface skin-layer formation that reduces insulin permeation in a thresholded, pancreas-like manner under physiological conditions (Figure 2). *In vitro* measurements showed glucose-dependent insulin release profiles consistent with the proposed diffusion-control mechanism while maintaining insulin activity. *In vivo*, the implanted device responded to nutritional perturbations and improved glycemic control in both insulin-deficient and insulin-resistant mouse models, with durability of at least 3 weeks. Based on the strategy of PBA-based synthetic glucose-responsive gel, an enzyme-free polymeric microneedle-array patch was also developed as a closed-loop, ‘on-skin’ artificial pancreas that achieves glucose-responsive insulin release without electronics, enzymes, or nanoparticles [49]. Similarly, glucose-responsive gel systems have been fabricated into microneedle patches for continuous glycemic control [50,51]. For example, Gu *et al*. integrated hypoxia-responsive vesicles, triggered by the enzymatic oxidation of glucose, or PBA-modified polymers within painless microneedles [50]. These patches have demonstrated rapid, closed-loop insulin delivery in type 1 diabetic mouse models, representing a significant step toward clinical translation. These glucose-responsive systems indicate substantial materials-level robustness for practical handling and storage. Their design addresses both sustained and acute glucose-responsive insulin delivery within a single microneedle-format platform, representing a material solution to two key technical challenges in transdermal insulin delivery. This long-acting, on-demand microneedle technology is likely to be a next-generation diabetes therapy that is stable, safe, economically efficient, and relatively insensitive to user compliance while enabling continuous glycemic control. Thus, glucose-responsive hydrogels offer a promising materials platform for low-burden insulin therapy in patients with diabetes by enabling autonomous, glucose-triggered dosing that can reduce monitoring demands and improve glycemic control.

### 2.2. Protein-responsive hydrogels

**Figure 3.**
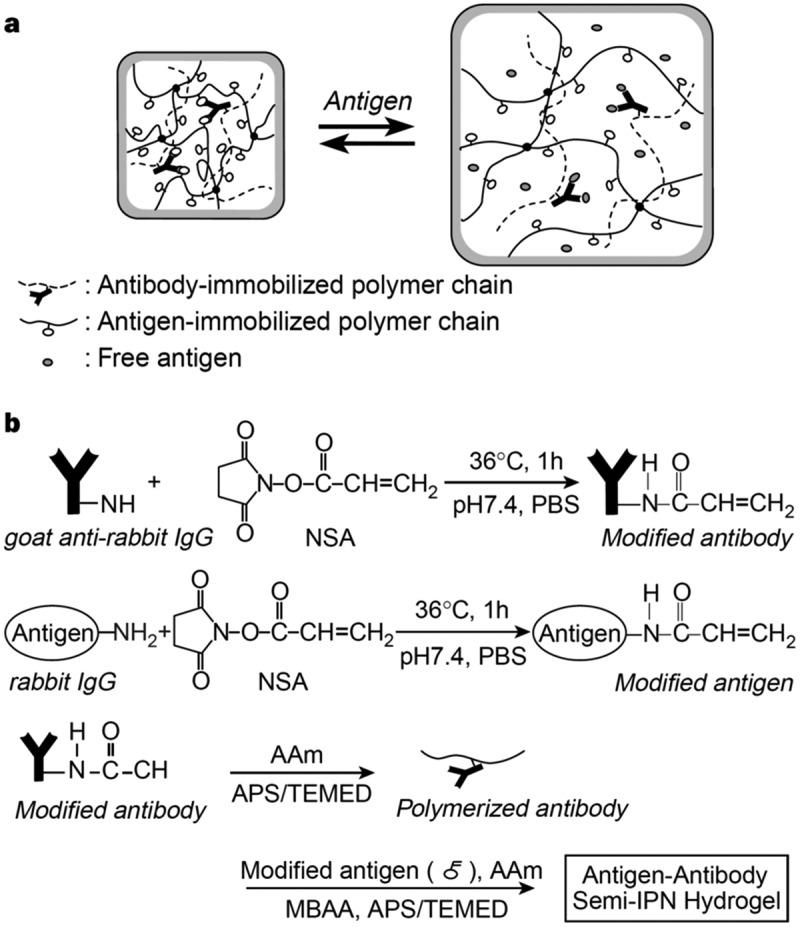
Strategy for the preparation of an antigen-responsive hydrogel. (a) Mechanism for the swelling of an antigen-antibody semi-IPN hydrogel in response to a free antigen. (b) Synthesis of the antigen-antibody semi-IPN hydrogel. Reproduced from Ref. [52] with permission from Springer Nature. Copyright 1999 Springer Nature.

Biomolecularly stimuli-responsive hydrogels have been designed using the concept of converting biochemical recognition directly into macroscopic structural change. For example, semi-interpenetrating polymer network (semi-IPN) hydrogels with antigen-antibody complexes as dynamic crosslinks undergo a change in volume in response to a target antigen [52–55]. The antigen-responsive hydrogels were prepared by copolymerizing a modified antigen (anti-rabbit IgG) with acryloyl groups, acrylamide (AAm), and *N,N’*-methylenebisacrylamide (MBAA) in the presence of an antibody-conjugated polymer (goat anti-rabbit IgG-modified linear polyacrylamide) forming an antigen-antibody complex. In the resulting hydrogels, the antigen-antibody complex linked with PAAm linear polymers and networks functioned as dynamic, non-covalent crosslinkers. In the absence of the free target antigen, the antigen-antibody complex forms a stable crosslink, maintaining the hydrogel in a shrunken state with a high effective crosslinking density (Figure 3). Upon exposure to the free target antigen (native rabbit IgG), a competitive ligand exchange of the antigen-antibody complex occurs within the hydrogel. The free antigen, having a higher diffusivity and relatively higher affinity, displaces the polymer network-bound antigen from the antibody binding sites. Therefore, the free antigen induces the dissociation of the antigen-antibody complexes acting as dynamic crosslinks within the network, thereby decreasing the crosslinking density. Consequently, the hydrogel undergoes thermodynamically driven swelling in response to a target antigen. This antigen-responsive swelling is not only a passive response but also an active actuation driven by the free energy change of the binding event. The response kinetics were relatively slow due to the restricted diffusion of the bulky antigen (150 kDa) within the polymer networks. To address this transport limitation, the semi-IPN architecture was rigorously optimized. Controlling the molecular weight of the linear antigen-polymer chains and the mesh size of the hydrogel network successfully facilitated the diffusion of the target antigen and a model drug [54,55]. The introduction of the semi-IPN structures enabled fully reversible changes in the volume of the antigen-responsive hydrogels in response to stepwise changes in the antigen concentration. Removing the free antigen shifts the equilibrium back, shrinking the gel back to its original state because the polymer network-bound antigen rebinds with the PAAm-modified antibody. This work was the first to demonstrate that highly specific biological recognition could be converted to reversible macroscopic structure changes for regulating drug release.

**Figure 4.**
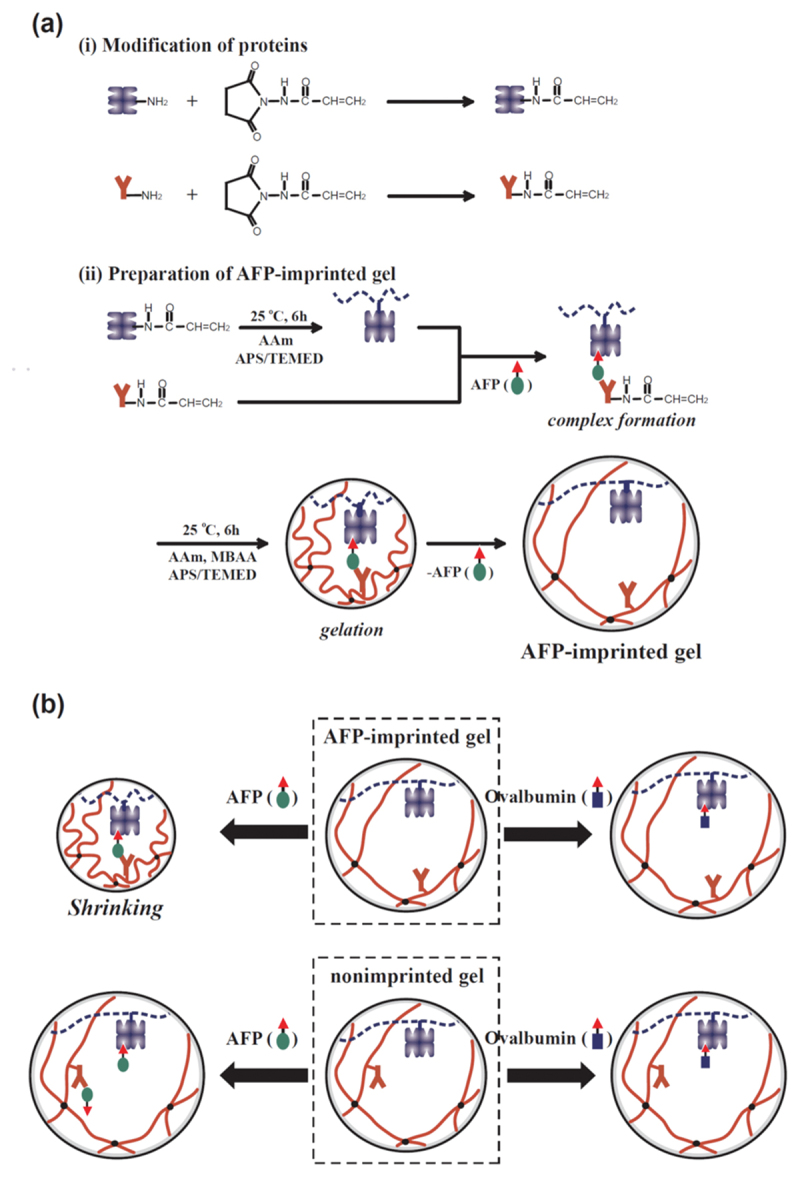
Strategy for the preparation of tumor marker-responsive hydrogels by biomolecular imprinting. (a) Synthesis of the tumor marker-imprinted hydrogels using lectins and antibodies as ligands for template glycoprotein molecules (tumor-specific marker AFP). (b) A schematic representation of glycoprotein-responsive behavior of tumor marker-imprinted and nonimprinted hydrogels. Reproduced from Ref. [56] with permission from National Academy of sciences. Copyright 2006 National Academy of sciences.

To address the complexity of cancer biomarkers, which often require multipoint interaction to achieve clinically relevant specificity, a biomolecular imprinting strategy was proposed using biomolecules as ligands [56,57]. An important target biomolecule is α-Fetoprotein (AFP), which is a glycoprotein widely used as a specific marker for hepatocellular carcinoma. As AFP contains both a peptide chain and a specific sugar chain, a dual-recognition approach is necessary to distinguish it from other serum proteins. AFP-imprinted hydrogels were strategically synthesized by biomolecular imprinting using two distinct ligands: a lectin ConA that specifically recognizes the sugar chain, and an antibody that recognizes the peptide chain. After polymerizable acryloyl groups were introduced to the lectin and antibody, they were copolymerized with acrylamide (AAm) and a minute amount of chemical crosslinker in the presence of the target AFP as a template. This process can effectively immobilize the lectin and antibody in a spatial arrangement complementary to the AFP molecule, creating a memory of the template’s shape and functionality. The resulting AFP-imprinted hydrogel shrank specifically in response to AFP (Figure 4). In typical responsive hydrogels, analyte binding often causes swelling due to osmotic pressure. Therefore, nonimprinted hydrogels showed negligible response or slight swelling in the presence of AFP because AFP forms a complex with either lectin or antibody ligands but forms no ternary lectin-AFP-antibody complex. In contrast, in the AFP-imprinted hydrogels, the formation of the ternary lectin-AFP-antibody complex, which acts as a dynamic crosslink, increases the crosslinking density of the hydrogel networks. As a result, the AFP-imprinted hydrogel can shrink only when both lectin and antibody ligands recognize the target AFP simultaneously. It should be noted that the specificity of the AFP-imprinted hydrogels is remarkable. This synergistic AFP recognition demonstrates that combining multiple weak or moderate interactions within a polymer network can yield high-affinity, specific materials that mimic the multicomponent binding sites of natural receptors.

### 2.3. Stimuli-responsive hydrogels using host-guest interactions

**Figure 5.**
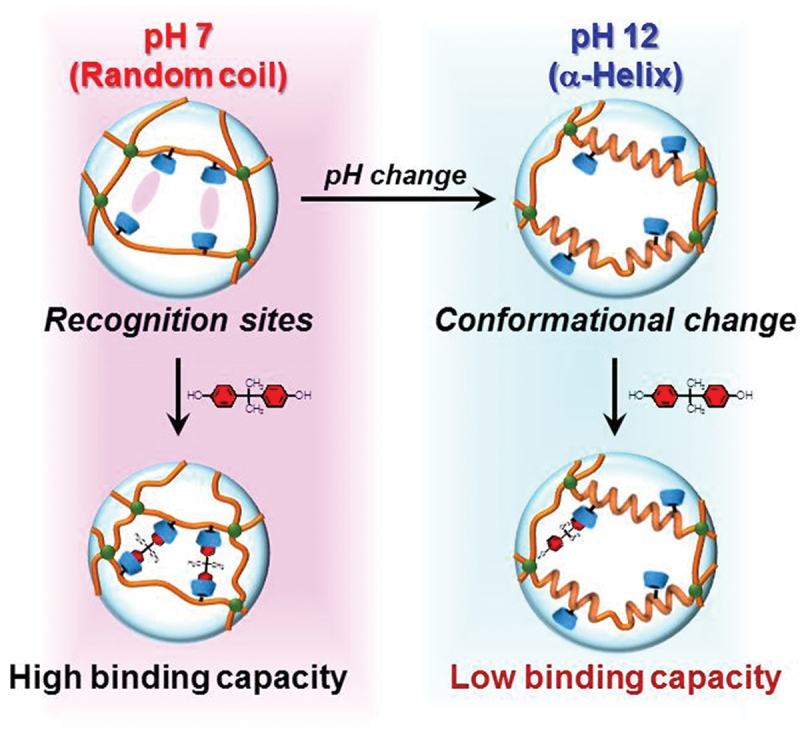
Schematic illustration for pH-responsive regulation of the binding capacity of the molecularly imprinted CD-PLL hydrogel by the conformational change from random coil to α-helix. Reproduced from Ref. [62] with permission from American Chemical Society. Copyright 2017 American Chemical Society.

**Figure 6.**
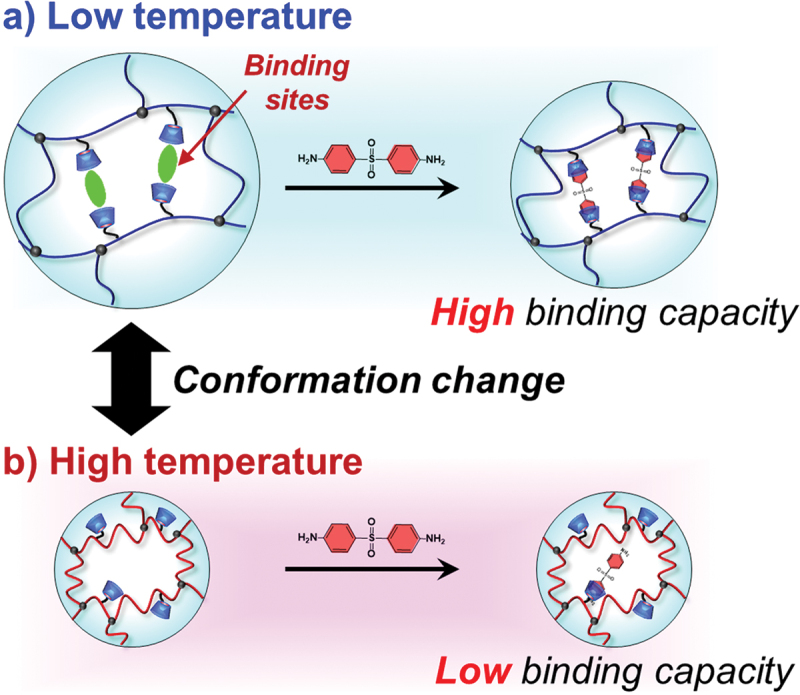
Schematic illustration of the regulation of the drug-binding capacity by a conformational change of a temperature-responsive MIP hydrogel with dynamic molecular binding sites at low (a) and high (b) temperatures. Reproduced from Ref. [63] with permission from Royal Society of chemistry. Copyright 2022 Royal Society of chemistry.

Supramolecular chemistry, particularly molecular recognition based on host-guest interactions, provides a powerful design tool for stimuli-responsive hydrogels, because reversible and selective noncovalent binding can be programmed into the network to translate an external signal into controlled changes in crosslinking density, permeability, and mechanical properties of the hydrogels. For example, Harada *et al*. converted the molecular recognition events to macroscopic assembly of hydrogel blocks [27]. They prepared separate hydrogel blocks functionalized with cyclodextrin (CD) as a host molecule and adamantane as a guest molecule. These macroscopic blocks were spontaneously and selectively assembled into larger structures through the formation of inclusion complexes between CD and adamantane at the interfaces. This macroscopic recognition was visible to the naked eye and highly selective. Namely, CD-containing hydrogels only adhere to adamantane-containing hydrogels, not to other CD-containing hydrogels. Harada *et al*. further advanced host-guest chemistry to create photo-responsive artificial muscle [58] and self-healing materials [59,60]. Self-healing hydrogels that can repair mechanical damage were prepared by using reversible inclusion complexation between CD and guest molecules as a dynamic crosslink. When the hydrogel is cut, the dynamic host-guest interactions at the fractured surface re-associate, restoring the material’s integrity and mechanical strength. The concept using host-guest interactions was extended to the combination of stimuli-responsive hydrogels and molecular imprinting. For example, after CDs were introduced as ligands into a poly(L-lysine) (PLL), molecularly imprinted polypeptide hydrogels were prepared by crosslinking of the resulting CD-PLL in the presence of a template bisphenol A (BPA) [61,62]. A pH increase induced the conformational change of the molecularly imprinted CD-PLL hydrogels from a random coil to an α-helix. The BPA-binding capacity of the hydrogels at neutral pH was higher than that at basic pH (Figure 5). This means that the molecular binding capacity of polypeptide hydrogels can be regulated by the pH-responsive change of the polypeptide conformation. Similar to pH-responsive hydrogels, the molecular binding capacity of the temperature-responsive hydrogels can be regulated by the conformational change in response to temperature changes. For example, temperature-responsive molecularly imprinted (MIP) hydrogels were prepared using diaminodiphenyl sulfone (dapsone) as a template model drug, CD as the ligand, and PNIPAAm as the primary chain so that the binding capacity can be switched by the coil-globule transition (Figure 6) [63]. Both MIP and nonimprinted (NIP) hydrogels shrank sharply near 37°C, which is close to body temperature. A larger amount of model drug dapsone was adsorbed into the MIP hydrogels than the NIP hydrogels below the transition temperature because molecular imprinting organizes CD ligands to form effective binding sites. When heated above the transition temperature near 37°C, those binding sites were collapsed by network shrinkage, and drug adsorption decreased even though hydrophobic interactions would ordinarily strengthen. These results indicate that the drug-binding capacity of the MIP hydrogels can be regulated by temperature-responsive conformational change. As a result, the MIP hydrogel effectively suppressed drug release below body temperature, but released rapidly above the body temperature, in contrast to drug release behavior from the usual PNIPAAm hydrogel, whose shrinking at a high temperature suppresses drug release. Molecularly imprinted hydrogels composed of temperature-responsive polymer chains enable a practical temperature-triggered off-on regulation of drug release based on the smart reservoir concept. Thus, the combination of molecular imprinting and stimuli-responsive networks can create dynamic molecular binding sites that can be regulated by conformational changes in response to stimuli like pH and temperature, mimicking dynamic molecular recognition of proteins.

**Figure 7.**
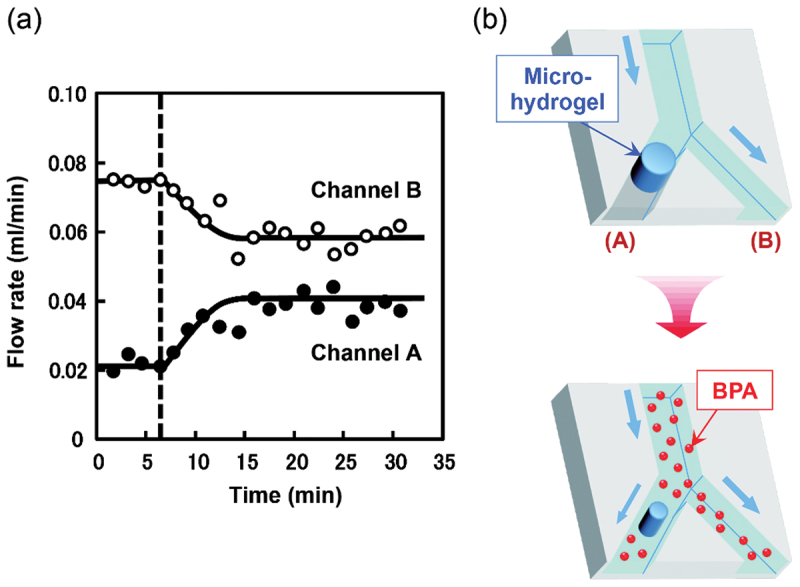
(a) Flow rate change of channel A (closed circle) with a BPA-imprinted micro-hydrogel and channel B (opened circle) without micro-hydrogel as a function of the time when deionized water and an aqueous BPA solution (120 µg mL^−1^) were flowed through the microchannel at a rate of 0.1 mL min^−1^. (b) Schematic of the flow change induced by responsive shrinkage of the BPA-imprinted micro-hydrogel in the Y-shaped microchannel. Reproduced from Ref. [64] with permission from wiley-VCH. Copyright 2015 wiley-VCH.

Integrating molecular recognition gels into microdevices allows for autonomous flow control, a key component of Lab-on-a-Chip systems. Bisphenol A (BPA)-responsive hydrogels with a micro-size were fabricated within microchannels by *in situ* photopolymerization techniques using a fluorescence microscope [64]. The micro-sized hydrogel with CD ligands was positioned to act as a valve in the microchannel. When the fluid contained trace amounts of BPA, the micro-sized hydrogel with CD ligands recognized the target BPA and shrank rapidly (Figure 7). This shrinkage of the hydrogel valve opened the flow channel, allowing the fluid to pass. Conversely, in the absence of BPA, the hydrogels swelled and blocked the flow. This autonomous regulation demonstrates the potential for creating self-powered environmental monitoring systems that mechanically sort samples based on chemical content.

### 2.4. Molecularly stimuli-responsive sol-gel transition

Formation and dissociation of biomolecular complexes, such as DNA duplexes and antigen-antibody complexes, are also useful tools in developing sol-gel phase transition in response to a target molecule. DNA offers unparalleled programmability for recognition because it enables sequence-addressable and orthogonal recognition for complementary DNA due to the strict Watson-Crick base pairing rules. Willner *et al*. have constructed DNA hydrogels that undergo sol-gel transitions triggered by specific aptamer-ligand binding [65]. These hydrogels function as biological computers capable of executing Boolean logic operations (AND, OR, NAND). For instance, a DNA-based hydrogel can be formed by the crosslinking of two different DNA strands. The DNA-based hydrogel can be programmed to form and/or dissolve only when specific DNA inputs are present simultaneously (AND and OR gates) [66]. This capability allows for the design of smart drug delivery systems that minimize side effects by targeting only cells that meet complex physiological criteria. Willner *et al*. also developed DNA hydrogels with shape-memory properties regulated by pH and metal ions, e.g. using i-motif sequences [67]. Target-responsive hydrogels with DNA aptamers have been designed for controllable release [68–70]. When the aptamer-functionalized DNA chains capture a target molecule such as adenosine with high specificity, the hydrogels were dissolved because of the dissociation of the duplex between the DNA aptamer and DNA strand linked with polyacrylamide [68]. Furthermore, a biomolecularly stimuli-responsive sol-gel transition system was also developed using tetra-poly(ethylene glycol) (Tetra-PEG) polymers conjugated with avidin and biotin [71]. The highly specific avidin-biotin complex acts as a dynamic and reversible crosslink, forming a hydrogel that can be dissolved instantly upon addition of free biotin. These works demonstrate that biomolecular complexes enable us to fabricate molecularly stimuli-responsive sol-gel transition systems with a programmable material life-cycle.

## 3. Particles, microgels, and nanogels with molecular recognition

### 3.1. Molecularly imprinted polymer particles

**Figure 8.**
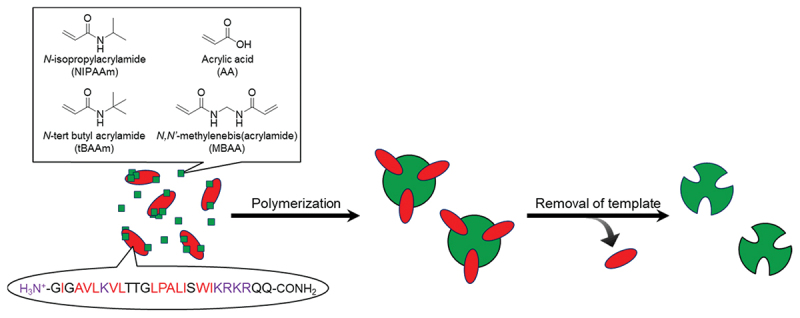
Preparation of peptide-imprinted polymer nanoparticles by the copolymerization of *N*-isopropylacrylamide, acrylic acid, *N*-tert butyl acrylamide, and *N,N’*-methylenebisacrylamide in the presence of a template peptide [73,74].

The miniaturization of polymeric materials with molecular recognition into micro- and nanoparticles offers significant advantages because their high surface-to-volume ratios enable rapid binding and response kinetics. Polymer particles provide a particularly useful platform for molecular recognition because many binding sites can be introduced on their wide surfaces. They have been widely used in sensing, separations, and biointerfaces because of not only their large surface area, but also responsive changes in dispersibility and optical properties. In practice, molecular recognition in polymer particles is rarely a single ‘lock-and-key’ interaction like those of enzymes. Instead, the affinity of the polymer particles usually emerges from the cooperative balance of hydrophobic interaction, electrostatic interaction, hydrogen bonding, and multivalent host-guest interaction, all constrained by crosslinking density and the polymer’s local segmental mobility. From a synthetic standpoint, polymer particles are generally prepared by emulsion polymerization, precipitation polymerization, inverse microemulsion/miniemulsion polymerization, and solid-phase (template-immobilized) imprinting. The preparative methods of polymer particles need to be chosen to control particle size, dispersity, and the spatial placement of recognition sites near the particle surfaces, where mass transport penalties are minimal. Recently, molecular imprinting technology has been widely used to prepare polymer particles with molecular binding sites for fabricating biosensors and separation systems [72]. Many researchers have also focused on polymer particles with molecular recognition for not only static adsorption but also tunable, environment-dependent bindings. For example, Hoshino *et al*. proposed plastic antibody concepts in which recognition is encoded as a nanoscale, crosslinked polymer matrix rather than a folded biomacromolecule [73–76]. They prepared peptide-imprinted polymer nanoparticles as synthetic affinity reagents by polymerizing functional monomer combinations in the presence of the target peptide melittin in water at room temperature (Figure 8) [73,74]. The affinity and selectivity of the peptide-imprinted polymer nanoparticles depended on crosslinked nanostructures whose apparent binding affinity was comparable to that of natural antibodies. Importantly, this study demonstrates the concept that a crosslinked polymer nanoparticle can be used as a designed receptor, provided that composition, crosslinking, and template removal are controlled. By expanding the concept, polymer nanoparticles that can functionally neutralize a biomacromolecular toxin were synthesized using melittin as a stringent target even in complex biological media [75]. This study explicitly clarified the relationship between the monomer feed composition and neutralization constants, converting molecular recognition events to a functional readout rather than adsorption alone. The melittin-recognition of the polymer nanoparticles is attributed to their multivariate property, i.e. hydrophobic and ionic interactions, polymer density, and temperature-responsive behavior, which influence binding/neutralization strongly. This indicates that polymer particles can be optimized against a function, such as toxin inhibition, not merely an equilibrium binding number. The practical problem that needs to be overcome in imprinting hydrophilic peptides is how to orient binding sites toward the particle surface when polymerization is carried out in heterogeneous media. Using inverse microemulsion polymerization, the imprint peptide was oriented at the water/oil interface by appending fatty acid chains of controlled length to the peptide, and then the affinity was evaluated by QCM [76]. Template localization during polymerization translated into a higher density of accessible, surface-proximal recognition domains. These studies indicate that the affinity and selectivity of polymer nanoparticles with molecular recognition sites can be improved by the approach from not only monomer designs but also interfacial physics during polymerization.

Conventional solution-phase imprinting often suffers from binding site heterogeneity, residual template contamination, and complex, poorly standardized manufacturing workflows. To address these issues, a solid-phase strategy has been developed to produce molecularly imprinted polymer (MIP) nanoparticles by covalently immobilizing templates on glass beads, followed by polymerization and on-support affinity purification [77,78]. A different route to higher reproducibility and tighter binding-site distributions is to immobilize templates on a solid phase and let the solid support serve as both imprinting scaffold and affinity purification medium. The solid-phase strategy yields essentially template-free MIP nanoparticles with high affinity for the target molecule, such as melamine and trypsin, and more uniform binding characteristics, based on the full protocol from immobilization to purification/characterization, which can be completed in roughly one week. For example, a solid-phase method was developed to produce MIP nanoparticles as plastic antibodies by fixing a reusable template onto glass beads in an automated UV reactor [77]. This method can precisely regulate particle sizes from 30 to 400 nm and yields high-affinity MIP nanoparticles devoid of template contamination. The resulting MIP nanoparticles are suitable for analytes like melamine (*K*_d_ = 6.3 × 10^−8^ M), vancomycin (*K*_d_ = 3.4 × 10^−9^ M), peptides, and proteins, with binding uniformity ensured by affinity-based purification. Notably, the immobilized templates endure over 30 synthesis cycles without degradation. This results in cutting expenses and risks while supporting scalable automation. These stable MIP nanoparticles show antibody-like performance in selected assays while offering improved thermal/chemical stability.

**Figure 9.**
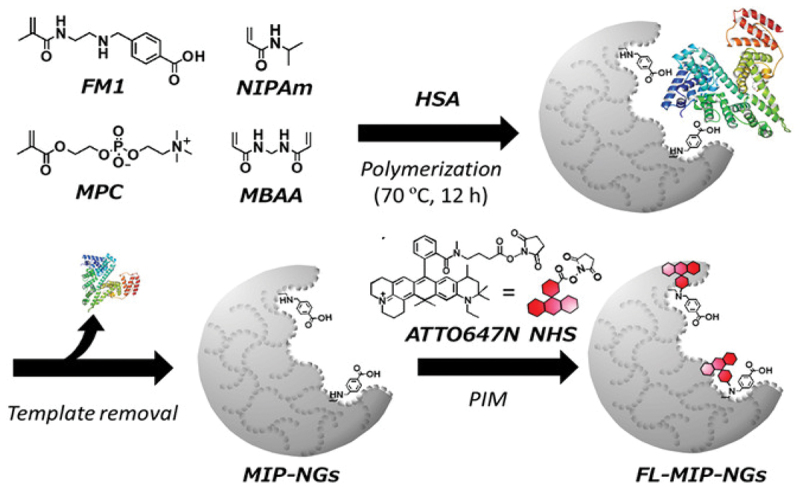
Schematic illustration of the preparation of the fluorescent-signaling MIP nanogels by molecular imprinting and postimprinting modification. Reproduced from Ref. [80]. Tsutsumi K. et al., Adv. NanoBiomed Res., 2021, 1, 2,000,079, licensed under creative commons attribution 4.0 (CC BY 4.0).

Recent work by Haupt *et al*. has extended molecular imprinting to fluorescent MIP nanoparticles for cellular imaging [79]. These MIP nanoparticles, as plastic antibodies, could stain cells and tissues with specificity comparable to biological antibodies but with superior stability. Fluorescent-signaling molecularly imprinted nanoparticles were also fabricated for selective protein detection by combining molecular imprinting with postimprinting modification (PIM), thereby enabling binding events to be transduced directly into a fluorescence readout [80]. Specifically, human serum albumin (HSA) was used as the model template to prepare HSA-imprinted nanoparticles *via* emulsifier-free precipitation polymerization using a bivalent functional monomer, 4-[2-(*N*-methacrylamido)ethylaminomethyl]benzoic acid, together with NIPAAm, 2-methacryloyloxyethyl phosphorylcholine (MPC), and MBAA. After template removal, a fluorescent dye was conjugated to residual amine sites by PIM to yield approximately 20 nm fluorescent MIP nanoparticles that retain high-affinity binding (Figure 9). The resulting sensing concept achieves sensitive and selective HSA detection, which shows markedly reduced cross-reactivity versus reference proteins and provides a quantitative readout even in diluted clinical serum with good agreement to a conventional assay. This demonstrates a general strategy for creating signaling-capable molecularly imprinted nanoparticles for bioanalysis.

### 3.2. Responsive micro/nanogels with molecular recognition sites

A microgel is defined as a gel particle with a diameter of approximately 0.1 μm to 100 μm. Microgels possess attractive structures and properties of both hard particles and bulk hydrogels, and behave as colloidal dimensions rather than macroscopic hydrogels [81]. For example, they have crosslinked network structures, but their colloidal dimensions shorten diffusion lengths, allowing rapid swelling/deswelling. In microgels, molecular recognition can be effectively and rapidly transduced into detectable signals, i.e. changes in size, dispersibility, and optical properties. Reversible noncovalent interactions, such as hydrogen bonds, ionic interactions, and host-guest interactions, play an important role in the molecular recognition of the microgels. The binding capacity of the microgels with molecular recognition is strongly influenced by their network structures. The amplification of molecular binding into macroscopic changes in the structure depends on the crosslinking density, osmotic pressure, and stability in aqueous media. In general, microgels have been synthesized by heterogeneous polymerizations such as emulsion polymerization, precipitation polymerization, inverse emulsion/miniemulsion polymerizations, and templated hybridization with inorganic domains. The synthetic methods for microgels permit control of their size, crosslinking density, and the distribution of recognition sites. In the effective design of microgels with molecular recognition sites, molecular binding induces changes in local crosslinking and charge balance, and then the microgel network responds as a collective behavior rather than a set of independent binding sites. For example, a series of glucose-responsive microgels was prepared by aqueous free-radical precipitation polymerization using *N*-alkylacrylamide derivatives as temperature-responsive moieties and PBA as a glucose-recognition site [82]. As a function of network composition, the resulting microgels displayed two opposing responses to increasing glucose levels, either volumetric swelling or shrinking, arising from distinct binding modes. Both response types of the microgels are relevant to glucose sensing and insulin/drug delivery. In the swelling mode, complexation of glucose with a single boronate receptor increases hydration/osmotic pressure and promotes solute transport by diffusion through the expanded network. On the other hand, in the shrinking mode, glucose interacts with two boronate units to generate additional effective cross-links that induce collapse. Such glucose-responsive behaviors of the microgels enable higher-selectivity sensing and intelligent valve behavior in microfabricated delivery devices. The glucose-triggered directionality can be programmed by rational selection of the alkylacrylamide and boronate components, as well as the cross-linker and local basic functionality, e.g. introducing a proximal amine to depress the boronic-acid pKa. This work provides some general rules to tune the swelling/shrinking behavior of glucose-responsive microgels.

**Figure 10.**
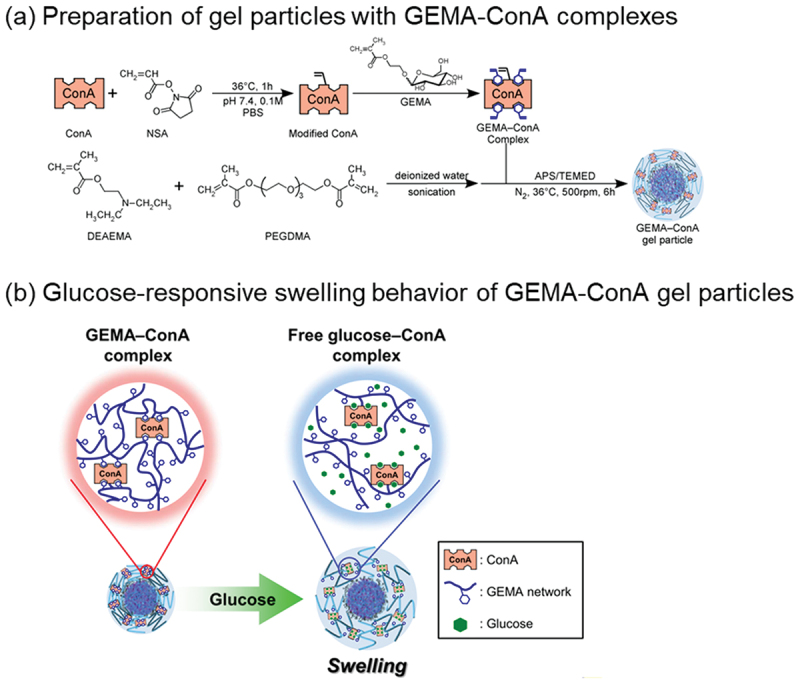
(a) Preparation of bioconjugated gel particles containing GEMA – ConA complexes by surfactant-free emulsion polymerization. (b) Schematic of glucose-responsive behavior of GEMA – ConA gel particles. Reproduced from Ref. [100] with permission from Elsevier. Copyright 2012 Elsevier.

Strategic design of glucose-responsive micro/nanogels aims to overcome the limitations of glucose-responsive hydrogels with macroscopic size to achieve effective size or volume modulation in response to glucose levels [83,84]. Traditional glucose-responsive platforms often combine GOx with pH-responsive polymers [37,38,85–87]. For instance, Volpatti *et al*. developed acetalized dextran nanoparticles encapsulating insulin and GOx [88]. The oxidation of glucose by GOx lowers the local pH, triggering the degradation of the nanoparticles and subsequent insulin release. These particles demonstrated effective glucose-responsive regulation of insulin release in type 1 diabetic mouse models. Similarly, Zhao *et al*. utilized GOx-polymer nanogels to modulate hydrogen peroxide production for melanoma therapy, showing enhanced antitumor efficacy [89]. Alternatively, fully synthetic systems for glucose-responsive insulin release have been developed by combining the glucose-recognition function of PBA derivatives with temperature-responsive polymers [45,46,90,91]. Hoare and Pelton synthesized PNIPAAm-based microgels functionalized with PBA, which exhibited glucose-dependent swelling and charge inversion under physiological conditions [92]. Xing *et al*. analyzed the swelling kinetics of NIPAAm-PBA microgels [93], and Guo *et al*. developed fluorescent nanogels *via* RAFT polymerization that release encapsulated insulin in response to glucose concentration [94]. While these systems hold potential for treating diabetes [95–98], further research is required to address reproducibility and toxicity issues for clinical translation [99]. Another approach involves modulating the microgel size *via* dynamic crosslinking [15]. By incorporating molecular complexes, such as carbohydrate-lectin complexes and antigen-antibody complexes, as crosslinkers, gels can be designed to swell or shrink upon the recognition of target molecules [40–42,52–55]. Therefore, surfactant-free emulsion polymerization was used to prepare glucose-responsive microgels with GEMA-ConA complexes as dynamic crosslinks. The resulting microgels exhibited rapid swelling in response to glucose because glucose induced the dissociation of the GEMA-ConA crosslinks by complex exchange with GEMA (Figure 10) [100].

Molecularly imprinted hybrid microgels were designed to serve as optical glucose-indicators that enable highly sensitive and selective glucose monitoring in complex media under physiological pH conditions [101]. The optical glucose-indicator was fabricated by *in situ* immobilization of Ag nanoparticles (AgNPs) within molecularly imprinted glucose-responsive microgel templates bearing phenylboronic acids. Confining AgNPs in the immediate vicinity promoted efficient plasmonic coupling. The resulting gel-actuated and glucose-tunable coupling behavior of the embedded AgNPs in both phosphate buffer and artificial tear fluid exhibits an obvious color transition from yellow to red as glucose increases from 0 to 20.0 mM, permitting instrument-free visual readout. With an optimized AgNP loading, the surface plasmon resonance (SPR) signal shows minimal interference from major non-glucose components. Quantitative sensing in artificial tears with a root-mean-squared error of prediction of 13.7 μM (~0.2 mg/dL) over 0.1–20 mM (1.8–360 mg/dL) was achieved using the optical glucose-indicators. This means that they have practical applications for continuous glucose monitoring in clinical diagnostics and bioprocess settings. Thus, recognition chemistry and nanoscale photophysics can be integrated within a single colloidal device as a microgel-as-transducer.

**Figure 11.**
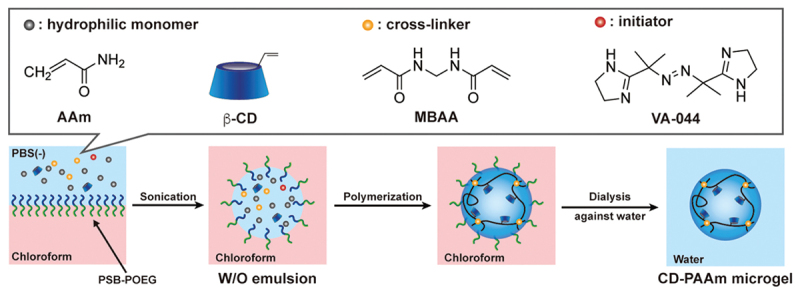
Schematic image of the preparation of the CD-PAAm microgel via inverse miniemulsion polymerization. Reproduced from Ref. [102]. Kawamura a, et al., 2026, 2,610,881, licensed under creative commons attribution 4.0 (CC BY 4.0).

Recently, Kawamura *et al*. reported a novel method to prepare molecularly stimuli-responsive microgels bearing recognition sites within a hydrophilic network by inverse miniemulsion polymerization using a water-soluble emulsifier [102]. An amphiphilic block copolymer as the emulsifier was synthesized *via* reversible addition-fragmentation chain transfer (RAFT) polymerization, consisting of a hydrophilic poly(sulfobetaine) block (PSB block) and a hydrophilic/oleophilic poly[oligo(ethylene glycol) methacrylate-*co*-2-(2’−methoxyethoxy)ethyl methacrylate] block (POEG block). The resulting poly(sulfobetaine)-*block*-poly[oligo(ethylene glycol) methacrylate-*co*-2-(2’−methoxyethoxy)ethyl methacrylate] (PSB-POEG) stabilizes the water-chloroform interface in a water-in-oil (W/O) emulsion. Using PSB-POEG-stabilized aqueous droplets as nanoreactors, inverse miniemulsion copolymerization of acrylamide (AAm), acryloyl-modified CD, and MBAA yields CD-conjugated PAAm (CD-PAAm) microgels with diameters of approximately 150 nm (Figure 11). The resulting CD-PAAm microgels shrank rapidly in the presence of BPA because BPA promotes the formation of CD-BPA-CD complexes that acted as dynamic crosslinks. The synthetic method provides a versatile platform for designing rapidly molecule-responsive microgels suitable for molecular sensing, separations, and drug delivery applications. Furthermore, organic-inorganic hybrid particles with CD ligands for a target BPA were developed to accelerate recognition. Silica particles coated with a CD-containing gel layer (CD-PAAm/SiO_2_) were synthesized by copolymerization of acryloyl-modified CD and AAm *via* surface-initiated atom transfer radical polymerization (SI-ATRP) on the silica particle surfaces. The CD-PAAm/SiO_2_ hybrid particles rapidly decreased their size upon exposure to BPA due to the formation of CD-BPA-CD crosslinks within the hydrogel shell [103]. Thus, responsive microgels can be designed by the introduction of ligands, which form complexes as reversible crosslinks with target molecules, into micro-networks swollen in aqueous media.

In addition, enzyme-responsive nanogels were designed by utilizing specific enzymatic reactions for drug release. Wang *et al*. prepared nanogels responsive to neutrophil elastase for tumor-targeted delivery [104], while Yang *et al*. created cytochrome c-loaded nanogels that selectively target CD44-positive cancer cells [105]. Lipase-responsive nanogels were also prepared to release antibiotics upon degradation by using bacterial lipase, offering a targeted approach for treating infections [106]. These works demonstrated that enzymes can be used as useful tools in designing micro/nanogels for medical applications. Even though micro/nanogels with molecular recognition still require future research work into possible applications, they are likely to become quite important materials for precision medicine in the future.

### 3.3. Self-assembled nanogels with molecular recognition sites

**Figure 12.**
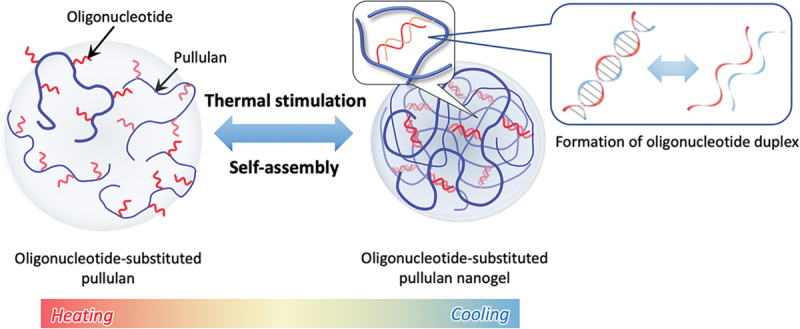
Schematic illustration of the formation of oligonucleotide-grafted pullulan nanogels. Reproduced from Ref. [114]. Sawada S, et al., Polym J., 2026, licensed under creative commons attribution 4.0 (CC BY 4.0).

While strictly speaking, microgels refer to crosslinked polymer particles with diameters ranging from 0.1 to 100 µm, the term nanogel is applied to similar crosslinked nanoparticles with diameters typically below 100–200 nm. The distinction is primarily based on the hydrodynamic radius of the swollen particles. Nanogels extend the microgel concept into smaller length scales where soft confinement and dynamic association/dissociation can behave like proteins and other biomacromolecules [107,108]. The molecular recognition of nanogels often arises from multivalent hydrophobic domains, host-guest interactions like CD, or engineered degradable linkages that gate access to internal domains. Nanogels are frequently prepared by self-assembling of amphiphilic polymers or by crosslinking within nanoscale compartments. While hydrophobic interactions, ionic interactions, and host-guest interactions play an important role in forming and stabilizing the nanogel, they can also act as binding elements for molecular recognition. Nanogels can show selective complexation and stimuli-responsive release profiles that resemble regulated binding rather than passive loading [109–111]. For example, Akiyoshi *et. al*. reported that a dynamic nanogel formed from cholesteryl-group-bearing pullulan and -CD exhibited a heat-shock-protein-like function in which thermally denatured protein was selectively trapped while native protein was released [112]. The recognition element of the nanogels is not a single binding site but a reversible host-guest network that can reorganize depending on CD concentration and temperature, which modulates association/dissociation equilibria with the protein substrate. Importantly, the recovery of enzyme activity of heat-denatured carbonic anhydrase B was achieved by trapping within nanogels and releasing from them. This means that the nanogel behaved as an active chaperone mimic rather than as a carrier. This work demonstrated that the selectivity of the nanogels with CD ligands may be expressed as preferential binding to a conformational state (denatured and native states), unlike small-molecule recognition of molecularly imprinted polymer materials. In addition, an acid-labile cholesteryl pullulan derivative was synthesized by grafting vinyl ether-cholesterol substituents onto a 100 kDa pullulan backbone, yielding a self-assembling nanogel that remains stable under near-neutral conditions [113]. At pH 7.0, the nanogels displayed a hydrodynamic radius of 26.5 ± 5.1 nm, whereas acidification to pH 4.0 induced a ~ 135% increase in size, consistent with pH-triggered structural reorganization. Size-exclusion chromatography further indicated that, at pH 4.0, the acid-labile grafts underwent substantial cleavage, while negligible degradation was observed for a pH-stable analogue under acidic conditions or for the acid-labile system maintained at pH 7.0. Protein complexation was demonstrated using bovine serum albumin, which associated with the nanogel at pH 7.0 and was subsequently released upon lowering the pH. The results establish a stimuli-responsive, self-assembled nanogel carrier in which acid-catalyzed cleavage of the cholesterol-pullulan grafting moiety governs protein release kinetics. This supports a promising use of the self-assembled nanogels as a responsive protein delivery platform using pH-degradable moieties. Recently, self-assembled nanogels were also prepared by grafting very short oligonucleotides (4-mer and 6-mer) containing bridged nucleic acids onto the water-soluble polysaccharide pullulan (Figure 12) [114]. In the nanogels, duplex formation between grafted strands serves as the noncovalent crosslinks. The nanogel formation depends strongly on oligonucleotide chain length, sequence, and the number of bridged-nucleic-acid substitutions, with the assemblies exhibiting temperature-dependent association/dissociation behavior. This strategic design of nanogels uses the high duplex stability conferred by bridged nucleic acid chemistry, which enables robust hybridization even with unusually short strands and thereby facilitates the formation of nanogels below 100 nm. Flow-cytometry measurements indicate markedly enhanced cellular uptake when oligonucleotides are delivered as nanogel complexes rather than as free strands. This work established oligonucleotide-grafted polysaccharide nanogels as a tunable, temperature-responsive carrier platform for intracellular delivery of nucleic acids and nucleic-acid-conjugated cargos under aqueous, physiologically relevant conditions. Thus, the duplex formation of oligonucleotides as recognizable moieties is a useful tool for designing stimuli-responsive nanogels with molecular recognition for medical applications.

## 4. Interfaces for intelligent gating and sensing

### 4.1. Gating membranes

Membranes, films, and interfacial layers can amplify molecular recognition through their 2D structures. In bulk materials, signals induced by molecular binding are diluted into 3D structures with large volumes. On the other hand, at surfaces and interfaces, especially within pores or in membranes, films, and layers within an evanescent sensing field, small molecular binding events can produce disproportionately large functional outputs. For membrane applications, the engineering constraints become more severe. For example, long-term exposure to pressure, flux, and complex feeds results in compaction, shear-driven rearrangement, and cumulative fouling. Stimuli-responsive membrane concepts have therefore been developed not only for gating, but also for fouling mitigation through controlled surface reorganization and cleaning cycles [115,116]. In particular, the addition of molecular recognition and responsiveness to membranes must not compromise the permeability, mechanical properties, and manufacturability [115]. Processing strategies are also important in practical applications of membranes with molecular recognition because they govern their structures, properties, permeability, and selectivity. For example, de Vos *et al*. proposed a sustainable aqueous phase separation approach to prepare stimuli-responsive membranes, which allows control of the structures and properties [117]. In the general designs of membranes with molecular recognition, a robust support carries the load, while a highly designed thin surface layer provides high selectivity and dynamic behavior [115–118]. Recognition ability becomes viable when the surface layer is uniform, strongly attached, and chemically stable during the operation.

**Figure 13.**
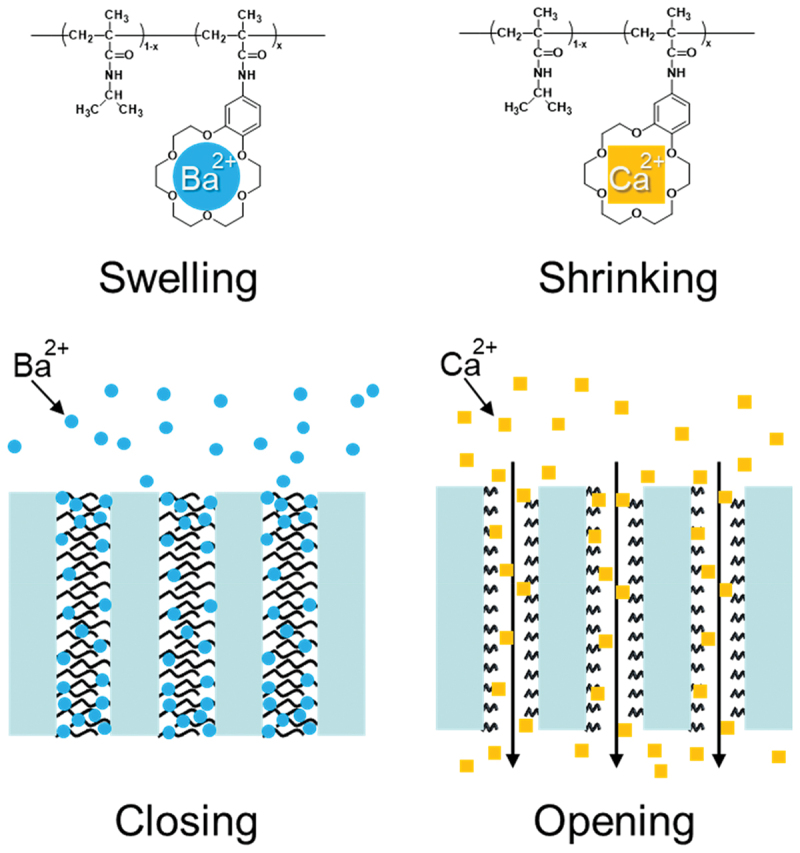
Ion-gating behavior of a porous polyehtylene membrane filled with a NIPAAm/benzo[18]crown-6-acrylamide copolymer synthesized by plasma graft copolymerization [119,120].

Gating membranes are representative membranes with molecular recognition because the responsive/recognizing polymers are precisely located at the surfaces and interfaces to govern the permeability of solutes. Yamaguchi *et al*. reported fast-response molecular recognition ion-gating membranes, in which a specific chemical signal altered the permeability by changing the state of polymer chains composed of temperature-responsive PNIPAAm and crown ether in or near the pore space [119–121]. The ion-gating membranes, in which porous polyethylene (PE) was filled with a NIPAAm/benzo[18]crown-6-acrylamide (BCAm) copolymer, were fabricated by plasma graft copolymerization. The LCST of the NIPAAm/BCAm copolymer shifts upon selective binding of ions, such as K^+^ or Ba^2+^, to crown ether enabled pore opening/closing *via* ion-responsive swelling-shrinking change at a constant temperature (Figure 13). The ion-gating membrane showed ~100 × higher flux in Ba^2+^ -containing solution than without Ba^2+^ and uniformly tuned its pore size from ~5 to 27 nm as a function of Ba^2+^ concentration (no change for Ca^2+^). This indicates utility for autonomous control of permeation flux and size-selective solute transport. Although the BCAm crown-ether receptors show a complexation constant about an order of magnitude lower than benzo[18]crown-6 and the fraction of complexed BCAm units remains below 5%, their complex formation still modulates the swelling-shrinking transition of the PNIPAAm chains. Differential scanning calorimetry revealed that increasing BCAm units raises the LCST and lowers the transition enthalpy, likely because ion-crown complexation disrupts hydration hydrogen bonding around hydrophobic moieties, thereby shifting the pore-opening temperature. Thus, combining ion-recognition of crown ether with temperature-responsive polymer chains provides design guidelines for stimuli-responsive graft copolymers and ion-gating membranes. The subsequent demonstration of controlled release in response to a specific ion signal showed that the concept can be operationalized as a device function [122]. These studies demonstrated that the gating membrane functions as a coupled chemo-transport system. Selectivity is expressed not only by preferential binding, but also by how binding perturbs the permeability, the local osmotic and conformational changes in confinement. In practical design of gating membranes, the grafting density, pore geometry, and polymer transition behavior must be tuned to match operating conditions.

### 4.2. Antifouling films

Two-dimensional polymer materials, including membranes, films, layers, and interfaces, provide important and useful environments for molecular recognition and binding events. In bulk hydrogels, microstructural heterogeneity can sometimes be averaged into an apparent binding constant or a macroscopic swelling ratio. By contrast, interfacial systems are governed by nanometer-scale parameters. For example, thickness, grafting density, segmental mobility, local hydration structure, and ionic partitioning determine whether a surface behaves as a passive sorbent for static binding or as an active element that modulates permeability, transport, adhesion, fouling resistance, optical response, and regenerability. The recent works on 2D polymer materials with molecular recognition sites have therefore focused on strategic designs, i.e. how to couple a selective interaction, such as host-guest, lectin-sugar, antigen-antibody, aptamer-protein, boronic acid-diol, and MIP cavity-template, to a controllable change in interfacial state, such as hydration, conformation, charge, porosity, permeability, or optical properties. Importantly, most recognition events at aqueous interfaces are competing with nonspecific adsorption. The competition is not only thermodynamic, but also kinetic. Proteins adsorbed at a surface undergo conformational changes like reorganization and spreading, often lowering free energy by maximizing contact area. Whitesides *et al*. used self-assembled monolayers (SAMs) as rigorous, composition-controlled model surfaces and then showed that protein adsorption can be interpreted in terms of water structure and interfacial free energy based on surface chemistry [123]. SAMs can provide methodological and conceptual information on protein adsorption because their surfaces are chemically well-defined. In addition, even when the nominal terminal group is the same oligo(ethylene glycol), the conformation and packing of the chains within the monolayer can decide whether a surface resists protein adsorption [124]. These results demonstrate that PEGylation is not a guarantee of nonfouling if the interface contains defects, collapsed segments, or sparse regions that act as adsorption nuclei. Whitesides *et al*. extended these concepts into a systematic structure-property relationship and showed that protein resistance depends on a subtle balance between interfacial energy, hydrogen-bonding, and chain organization. Their studies were useful in normalizing the idea that antifouling must be treated as a surface state rather than a single chemical identity [125]. Then, Jiang *et al*. addressed the molecular basis for nonfouling behavior in oligo(ethylene glycol)-terminated SAMs by linking protein adsorption behavior to how the interfacial layer structures water and limits favorable protein-surface contacts [126]. Thus, SAMs have been widely used as fundamental platforms not only for clarifying the relationship between chemical surface structure and protein adsorption but also for fabricating nonfouling surfaces.

**Figure 14.**
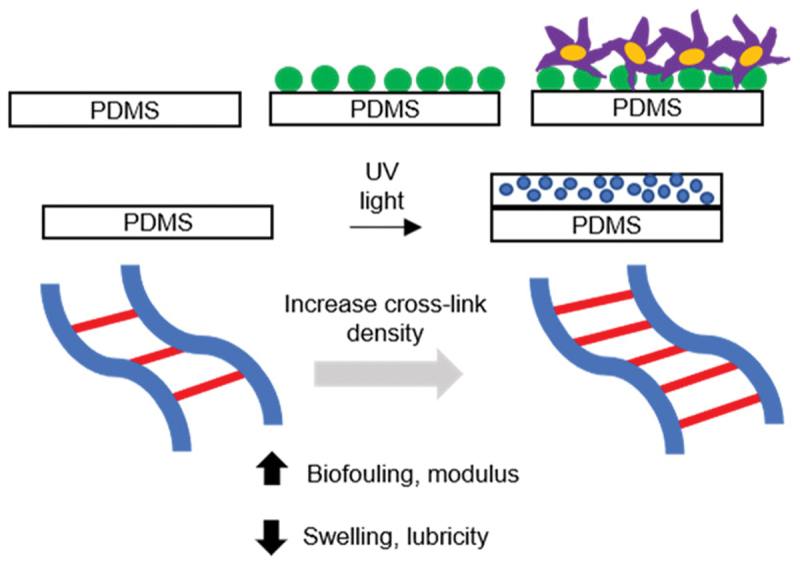
Preparation of zwitterionic polymer hydrogel films photografted onto polydimethylsiloxane (PDMS). Reproduced from Ref. [135]. Jensen M j, et al., ACS Biomater. Sci. Eng., 2021, 7, 4494–4502, licensed under the creative commons attribution 4.0 international license (CC BY 4.0).

Polymer brushes generalize the concept and methodology using SAMs into a solvent-swollen interfacial phase whose thickness, segment density profile, and hydration can be controlled. Polymer brushes are attractive platforms for molecular recognition because they are hydrophilic and give spatial control over where recognition events occur. Ligand molecules, such as proteins and host molecules, are displayed as receptors near the periphery to maximize accessibility, or are distributed within a hydrated layer to suppress nonspecific adsorption and stabilize conformation. Their physical and chemical structures have direct influences on association kinetics, dissociation kinetics, and regeneration behavior. In practice, polymer brush-based recognition layers frequently balance three competing demands, i.e. high hydration to minimize background, sufficient permeability to avoid diffusion-limited binding, and robust attachment to survive flow and repeated washing. The synthetic methods and characterizations regarding polymer brushes are summarized in several review papers [127–129]. Importantly, polymer brushes are not only coatings but also soft interfacial media, and their conformation can respond to salt, pH, temperature, and binding events. Such stimuli-responsive behavior of the polymer brushes can be applied to gate transport or modulate signal transduction [128,129]. The softness of the polymer brushes and layers is the most important feature because it can exhibit dynamic function. In designing polymer brushes with antifouling properties, not only PEG-based polymers but also zwitterionic polymers have been widely used for the formation of bioinert surfaces because they suppress nonspecific protein adsorption [130,131]. Jiang *et al*. emphasized that truly useful interfaces are not simply low-fouling and that they must also be functionalizable while maintaining hydration [132]. Antimicrobial and nonfouling behavior were also integrated in zwitterionic poly(carboxybetaine methacrylate) (pCBMA) brushes grafted from gold surfaces *via* ATRP, reducing long-term biofilm formation [133]. In addition, sensitive and specific protein detection in undiluted blood plasma was achieved using a zwitterionic poly(carboxybetaine acrylamide) (polyCBAA) brush surface [134]. The important point in surface modification using zwitterionic polymers is not only that nonspecific adsorption was reduced in a static incubation test, but also that baseline stability and specificity were improved even in aqueous media with various proteins. In particular, the strategy using zwitterionic polymers is useful in fabricating surface plasmon resonance sensors (SPR), quartz crystal microbalances (QCM), and related surface-sensitive methods because it reduces the need for sample pretreatment and enhances the signal-to-noise ratio. The influence of crosslinker concentration on the critical balance between mechanical stability and antifouling performance was investigated in zwitterionic polymer hydrogel films photografted onto polydimethylsiloxane (PDMS) (Figure 14) [135]. The hydrogel films with a broad range of crosslink densities significantly reduced protein adsorption, cell adhesion, and friction coefficients while maintaining an adequate compressive modulus, thereby establishing an optimal processing window for durable, low-fouling implantable biomaterials. Foreign-body reactions often degrade implantable biomedical devices by forming a dense collagen capsule that limits mass transport and electrical communication. However, ultra-low-fouling zwitterionic hydrogels suppress capsule formation for at least three months after subcutaneous implantation in mice and also enhance nearby angiogenesis, possibly by promoting pro-healing, anti-inflammatory macrophage phenotypes [136]. Their fascinating properties suggest that zwitterionic hydrogels are promising coatings or scaffolds for more biocompatible implantable devices and tissue engineering applications. Recent work to improve implantable devices and diagnostics emphasizes polymer biomaterials with strong resistance to protein adsorption by increasing surface hydrophilicity through physical and chemical surface modification such as physisorption, hydrogel formation, grafting, layer-by-layer assembly, and blending. Although PEG is still the representative bioinert polymer, alternatives, e.g. polyglycidols, poly(2-oxazoline)s, zwitterionic polymers, and so on, have increasingly become promising bioinert polymers for *in vitro* and *in vivo* protein adsorption resistance assessments using more complex protein media and more sensitive adsorption measurements [137]. Thus, antifouling is an important part of the recognition mechanism because it determines whether the specific interaction between a receptor and a ligand can be expressed against a low-noise background.

### 4.3. Polymer layers with molecular binding sites

Surface plasmon resonance (SPR) sensors have been powerful tools in biomedical and biochemical research because of their ability to quantitatively monitor dynamic interactions between biomolecules with high sensitivity [138–142]. The fundamental principle of the SPR sensor is based on detecting minute changes in the refractive index in the immediate vicinity of a gold surface, which are typically caused by the binding of an analyte to a ligand on the sensor chip. In conventional SPR sensors, ligands such as proteins and DNAs are chemically immobilized onto the sensor chip as a two-dimensional (2D) monolayer. However, the 2D configuration inherently limits the surface density of ligands, which in turn restricts the dynamic range and performance of the sensor. To overcome this limitation, the hydrogels that are three-dimensional (3D) crosslinked polymer networks swollen with water can be used as superior matrices for ligand immobilization. Their swollen networks not only allow for the rapid diffusion of solutes but also provide a flexible environment for polymer chains, thereby enhancing molecular recognition capabilities. Thus, as the 3D network enables immobilizing a large amount of ligands compared to flat 2D surfaces, the fabrication of hydrogel-coated sensor chips is a useful strategy for amplifying SPR signals. A practical example of this approach involves the fabrication of antibody-bearing hydrogel layers using SI-ATRP. This polymerization technique provides exceptional control over the molecular weight and thickness of the polymer layer by adjusting reaction parameters such as monomer concentration and polymerization time [143–145]. For example, after ATRP initiators were first attached to the SPR sensor chip surface, an acryloyl-modified antibody as a ligand was copolymerized with AAm to grow the hydrogel layer directly from the chip surface [146]. Scanning electron microscopy (SEM) observation confirmed that the layer thickness could be precisely tuned by varying the polymerization time. The resulting 3D hydrogel layers with antibodies demonstrated a superior binding capacity for the target antigen (rabbit IgG) compared to directly antibody-attached surfaces. The hydrogel layer sensor chips also maintained high specificity, showing negligible response to non-target proteins like bovine serum albumin (BSA). Kinetic analysis revealed that the hydrogel layer chip achieved an association constant (*K*_a_) of 1.3 × 10^5^ M^−1^, whereas the directly antibody-attached chip showed a lower affinity of 7.3 × 10^4^ M^−1^. It should be noted that the SPR signal intensity does not increase proportionally with layer thickness. The SPR signal intensity increased with layer thickness up to approximately 225 nm but declined thereafter. This phenomenon is governed by the decay length of the evanescent field, which only detects refractive index changes within ~300 nm of the gold surface in the SPR sensors. Consequently, although thicker hydrogel layers can hold more antibodies, binding events occurring beyond this detection limit do not contribute to the signal, and restricted diffusion in dense layers may further attenuate the response.

**Figure 15.**
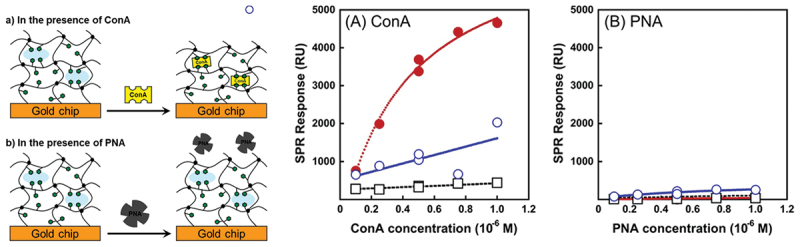
Molecularly imprinted hydrogel layer SPR sensor chips with lectin-recognition sites, which were prepared via surface-initiated atom transfer radical polymerization (SI-ATRP) combined with molecular imprinting, exhibited not only large SPR signal change in response to a target lectin but also inhibited nonspecific protein adsorption. Reproduced from Ref. [159] with permission from Springer Nature. Copyright 2018 Springer Nature.

Molecular imprinting is another powerful methodology in fabricating sensor chips, often described as creating ‘artificial locks’ for specific molecular ‘keys’ [147–150]. As described in the previous section, molecular imprinting involves polymerizing functional monomers around a template molecule, which is subsequently removed to form cavities that match the template’s size, shape, and chemical functionality. While standard molecular imprinting is well-established for small molecules, imprinting large and fragile biomolecules like proteins presents significant challenges due to their complex conformational requirements [6,151–156]. Therefore, hydrogel-based molecular imprinting strategies have been developed by using low crosslinking densities to provide the necessary structural flexibility for protein recognition. For instance, protein-imprinted hydrogel layers targeting concanavalin A (ConA) were formed on SPR chips using SI-ATRP [157]. A methacrylate monomer with a pendant glucose (GEMA) as the ligand, AAm as the matrix, and a minimal amount of MBAA as the crosslinker were copolymerized using ConA as a template by the SI-ATRP on the SPR chips. The removal of the ConA template after the network formation yielded a hydrogel layer with specific binding sites for ConA. The ConA-imprinted hydrogel layers with a thickness of ~289 nm exhibited a marked increase in SPR signal in response to ConA compared to nonimprinted hydrogel layers. The association constant for the imprinted hydrogel layer was determined to be 2.02 × 10^7^ M^−1^, significantly higher than the 5.26 × 10^5^ M^− 1^ for the nonimprinted hydrogel layers, validating the efficacy of the imprinting process. Similarly to the antibody-immobilized hydrogel layers, the signal response was found to decrease when the layer thickness exceeded the ~300 nm sensing depth of the SPR evanescent field. This means that optimizing layer dimensions is critical for maximizing sensor sensitivity. Not only the thickness but also the crosslinked network structure of the layer plays important roles in molecular binding performance. While the ‘brush-type’ imprinted layers without chemical crosslinking showed affinity levels similar to nonimprinted layers, the ‘gel-type’ imprinted layers with chemical crosslinking demonstrated a twofold increase in affinity [158]. This comparison between non-crosslinked and chemically crosslinked structures demonstrates the necessity of a crosslinked network to stabilize the spatial arrangement of functional groups within the imprinted cavities as molecular binding sites. Furthermore, suppressing non-specific adsorption can enhance selectivity effectively in complex biological environments. Integrating bioinert moieties, such as zwitterionic MPC, into the molecularly imprinted network is effective in fabricating sensor chips. For example, ConA-imprinted hydrogel layers synthesized with an MPC backbone exhibited high affinity for ConA while effectively resisting the binding of non-target lectins like peanut agglutinin (PNA) (Figure 15) [14]. The combination of molecular imprinting for specificity and bioinert MPC for background suppression represents a strategic approach to developing high-performance biosensors.

## 5. Conclusion

Research on polymer soft materials with molecular recognition initially emphasized static binding for separation, but the field has increasingly shifted toward responsive materials and systems that couple recognition to changes in structure or function. Incorporation of noncovalent interactions as molecular recognition moieties, including host-guest interactions, specific biomolecular interactions, and molecularly imprinted cavities, into stimuli-responsive polymer networks has enabled synthetic systems that have partially mimicked the allosteric regulation of natural proteins. The material architecture, ranging from bulk hydrogels to micro/nanoparticles and two-dimensional interfaces, significantly determines the performance and utility of these systems. Owing to the large volume changes and water-rich networks, bulk hydrogels have been widely explored for glucose-responsive insulin release and for self-regulating microfluidic valves that operate without external control. Miniaturization of these systems to particles and micro/nanogels generally enhances binding and response kinetics for targeted nanomedicine and intracellular drug delivery, as it shortens diffusion distances and improves access to recognition sites. Polymer interfaces, particularly functional membranes, thin films, and layers, are well suited to SPR biosensing and to stimulus-gated solute transport because recognition sites can be positioned directly at the interfacial region. Further progress will likely depend on whether biomimetic designs can reproduce not only selective binding but also the coupled structural and feedback responses characteristic of biological systems. More precise control over responsive behavior and over the spatial organization of recognition sites is likely to contribute to fabricating next-generations of diagnostic sensors, selective separation systems, and drug-delivery platforms with feedback capability.
